# Pyruvate Kinase Differentially Alters Metabolic Signatures during Head and Neck Carcinogenesis

**DOI:** 10.3390/ijms242316639

**Published:** 2023-11-23

**Authors:** Pei-Chun Huang, Ching-Wen Chang, Yu-Cheng Lin, Chang-Yi Chen, Tsai-Ying Chen, Lu-Te Chuang, Chung-Ji Liu, Chien-Ling Huang, Wan-Chun Li

**Affiliations:** 1 Institute of Oral Biology, College of Dentistry, National Yang Ming Chiao Tung University, Taipei 11221, Taiwan; peijyng@gmail.com (P.-C.H.); cychen.de10@nycu.edu.tw (C.-Y.C.); puddy0906@gmail.com (T.-Y.C.); 2 Graduate Institute of Metabolism and Obesity Sciences (GIMOS), College of Nutrition, Taipei Medical University, Taipei 11031, Taiwan; changc11@tmu.edu.tw; 3 Taipei Cancer Center, Taipei Medical University, Taipei 11031, Taiwan; 4 Department of Dentistry, College of Dentistry, National Yang Ming Chiao Tung University, Taipei 11221, Taiwan; ylin@nycu.edu.tw (Y.-C.L.); cjliu3229@gmail.com (C.-J.L.); 5 Oral Medicine Innovation Center (OMIC), National Yang Ming Chiao Tung University, Taipei 11221, Taiwan; 6 Department of Biotechnology and Pharmaceutical Technology, Yuanpei University of Medical Technology, Hsinchu 30015, Taiwan; ltchuang@mail.ypu.edu.tw; 7 Department of Oral and Maxillofacial Surgery, MacKay Memorial Hospital, Taipei 10449, Taiwan; 8 Department of Medical Research, MacKay Memorial Hospital, Taipei 10449, Taiwan; 9 Department of Health Technology and Informatics (HTI), The Hong Kong Polytechnic University (PolyU), Hung Hom, Kowloon, Hong Kong SAR, China; cl.huang@polyu.edu.hk

**Keywords:** head and neck cancer, glycolytic ATP, pyruvate kinase M2 form, metabolic reprogramming

## Abstract

During glycolysis, the muscle isoform of pyruvate kinase PKM2 produces ATP in exchange for dephosphorylation of phosphoenolpyruvate (PEP) into pyruvate. PKM2 has been considered as a tumor-promoting factor in most cancers, whereas the regulatory role of PKM2 during head and neck carcinogenesis remained to be delineated. PKM2 mRNA and protein expression was examined in head and neck tumorous specimens. The role of PKM2 in controlling cellular malignancy was determined in shRNA-mediated PKM2-deficient head and neck squamous cell carcinoma (HNSC) cells. In agreement with the results in other cancers, PKM2 expression is enriched in both mouse and human HNSC tissues. Nevertheless, PKM2 mRNA expression reversely correlated with tumor stage, and greater recurrence-free survival rates are evident in the PKM2^high^ HNSC population, arguing that PKM2 may be tumor-suppressive. Multifaceted analyses showed a greater in vivo xenografic tumor growth and an enhanced cisplatin resistance in response to PKM2 loss, whereas PKM2 silencing led to reduced cell motility. At the molecular level, metabolic shifts towards mitochondrial metabolism and activation of oncogenic Protein kinase B (PKB/Akt) and extracellular signal-regulated kinase (ERK) signals were detected in PKM2-silencing HNSC cells. In sum, our findings demonstrated that PKM2 differentially modulated head and neck tumorigenicity via metabolic reprogramming.

## 1. Introduction

It is widely appreciated that tumor cells possess distinct hallmarks to support their growth [1]. Among them, deregulated bioenergetics was considered as a potential link between tumor cells and their surrounding microenvironment [2,3]. Instead of genetic disturbance, a number of studies suggested that cancer might be originated from intrinsic/external metabolic imbalance, highlighting the crucial role of cell metabolism in controlling tumor malignancy [4]. While both cellular energy and biomass (e.g., nucleotides, amino acids, and lipids) are essential to support tumor cell survival [5], it is often observed that neoplastic progression would rely primarily on glucose fermentation during proliferation regardless of oxygen availability, known as the “Warburg effect” [6,7]. In the regard of energy demand, tumor cells preferentially utilize glycolytic ATP as energy sources, even though glycolysis provides less ATP compared to mitochondrial respiration [8]. To compensate lesser glycolytic ATP production, tumor cells evolved metabolically to produce lactate from glucose faster than the complete oxidation of glucose in the mitochondria by 10–100 times [9,10], making the ATP amount sufficient to support cell survival. In addition, a speedy glycolysis could maintain high levels of glycolytic intermediates to assist anabolic reactions of cellular biomass [11]. Indeed, by taking advantage of model analysis, a recent study revealed that stored glycolytic intermediates could possibly maintain energy supply at a level sufficient to maintain cell viability for up to 10 min under unfavorable environments [12], further providing an explanation for why increased glycolysis is often detected in highly proliferating cells throughout nature.

PhosphoGlycerate Kinase (PGK) and Pyruvate Kinase (PK) are ATP-generating glycolytic enzymes, as both also exhibit non-metabolic functions in different cell compartments [13]. At the metabolic basis, PGK and PK transfer an inorganic phosphate group to ADP from 1,3-bisphosphoglycerate (1,3-BPG) and phosphoenolpyruvate (PEP), respectively, to generate ATP in accompany with 3-phosphoglycerate (3-PG, by PGK) and pyruvate (by PK). As most studies confirmed that PGK has indispensable impact in the progression, metastasis, and drug resistance in many tumor types [14,15,16], the role of PK in regulating cancer malignancy is largely debated [17]. PK has four isoforms, including L, R, M1, and M2 [18], and a switch of PKM1 to PKM2 may play a major role in modulating tumorigenesis [19,20,21]. Interestingly, it was found that PKM2 in different cell compartments could be multifunctional by acting as metabolic controller and non-metabolic protein kinase in cytosol and serving as a transcriptional regulator when it is translocated into nucleus [22]. At the structure level, PKM2 is the only PK displaying functionally distinct tetrameric and dimeric forms. While PKM2 is in a tetrameric state, it serves as a glycolytic regulator to convert PEP to pyruvate; as the less-active dimeric PKM2 could be used as a switch for biosynthesis of cellular biomass during glycolysis [23]. The dimeric PKM2 is functionally versatile and could be detected either in nucleus to regulate gene expression, in mitochondrial outer membrane to maintain mitochondrial function, or in endoplasmic reticulum to inhibit endoplasmic reticulum stress [24]. In addition to oligomerization variance, post-translational modifications of PKM2, including phosphorylation, acetylation, oxidation, and prolyl hydroxylation, could also regulate PKM2 activity [25,26].

With complex regulatory cues in controlling PKM2 activity, it is not surprising that the role of PKM2 during tumorigenesis remained controversial. As PKM2 is enriched in most human cancers and is associated with poor clinical outcome [17], it is mostly reported that PKM2 serves as an oncogenic promoter to facilitate cell growth, metastasis, and chemoresistance by either regulating cellular metabolism or targeting different gene expression and signaling pathways as a nuclear transcription cofactor [22,27]. Furthermore, the circulating PKM2 level could be used as a diagnostic marker, along with other putative biomarkers, in cancer patients [28]. Nevertheless, previous investigations using genetically engineering mouse models showed discrepant results against the notion that PKM2 plays an oncogenic role in vivo. For instance, tumor formation is evident in a conditional knockout mouse model by deleting the PKM2 specific exon 10, implying that PKM2 may not be required for cancer cell proliferation [29]. Moreover, PKM2 knockout mice showed a high prevalence of spontaneous hepatocellular carcinoma, suggesting that PKM2 regulates systemic metabolic homeostasis and inflammation and control tumor development in a non-cell-autonomous mechanism [30].

As most studies used PKM2 expression as indicator of tumorigenic progression, the regulatory role of PKM2 in Head and Neck Squamous Cell Carcinoma (HNSC), the seventh most common type of cancer worldwide [31], remained to be clearly determined [32]. Early report showed that PKM2 expression is positively associated with major clinicopathological features and as an unfavorable prognostic marker in tongue squamous cell carcinoma and in oral squamous cell carcinoma (OSCC) [33,34,35]. At a molecular and metabolic basis, it was shown that (i) the PKM2/PKM1 ratio was higher in OSCC cells than in adjacent normal mucosal cells [36,37], and (ii) the EGFR-PKM2-β-catenin [38] and PAK2-c-Myc-PKM2 [39] cascades could support tumorigenesis via modulation of Warburg phenotype in HNSC cells. However, an extensive metabolic analysis in response to PKM2 alteration needs to be performed. In this study, multifaceted cellular and metabolic cues in shRNA-mediated PKM2-silencing HNSC cells were analyzed both in vitro and in vivo as well as in public HNSC datasets, in the hope to better define the PKM2-mediated metabolic regulatory cues during head and neck carcinogenesis.

## 2. Results

### 2.1. Differential Impacts of PKM2 in HNSC Progression and Prognosis

**Figure 1:**
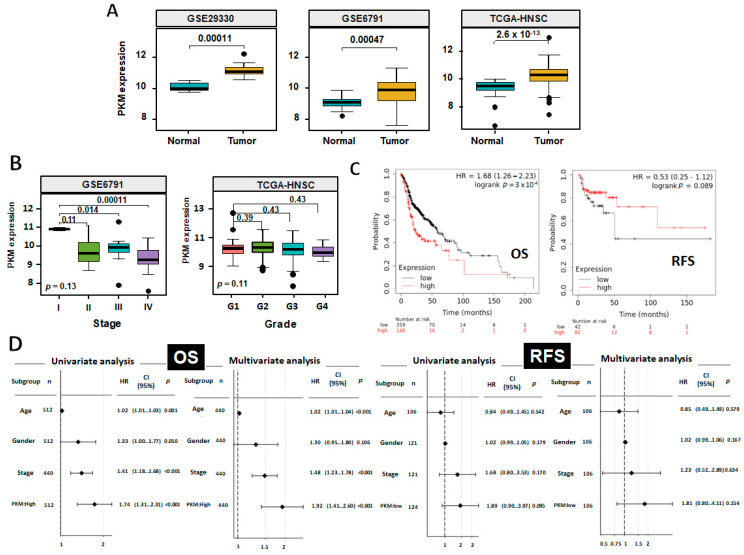
PKM2 Expression is Variable during HNSC Progression in Clinic. (**A**) Box-whisker plots showed the PKM2 mRNA levels in HNSC tumors and normal adjacent tissues from GEO and TCGA-HNSC databases. (**B**) Box-whisker plots showed the PKM2 mRNA levels in HNSC subjects, stratified by clinical stages from GSE 6791 and grades from TCGA-HNSC datasets. The boundary of the box closest to zero indicates the 25th percentile, a black line within the box marks the median and the boundary of the box farthest from 0 indicates the 75th percentile. Numbers above the comparisons indicate the *p*-values between groups. (**C**) Kaplan-Meier analysis for Overall Survival (OS) and Relapse-free Survival (RFS) rates in HNSC patients classified by PKM2 expression. The best cut-off values of 70,007 and 50,992 for OS and RFS, respectively, were used. (**D**) Univariate and multivariate Cox regression analysis for OS and RFS rates for TCGA-HNSC patients. PKM2 low population was selected as reference in OS analysis, whereas PKM2 high cells were selected as reference in RFS analysis.

**Figure 2:**
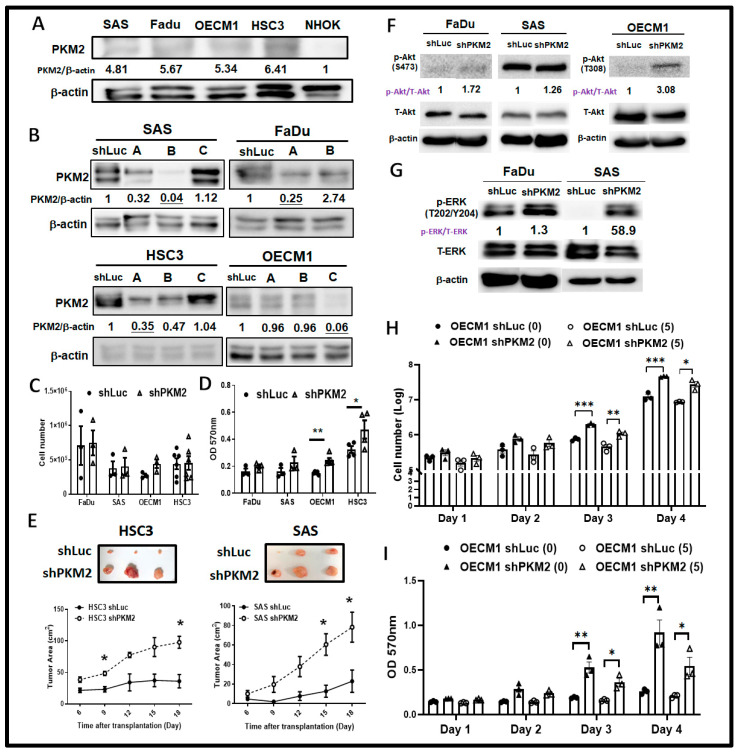
Differential Modulations for in vitro HNSC Cell Viability and in vivo Xenografic Tumor Growth in Response to PKM2 Loss. (**A**) Respective Western blot analysis for PKM2 protein expression in HNSC and NHOK cells. The representative ratio of PKM2 over internal control β-actin expression was quantified by Image J (v1.53t). (**B**) Western blot analysis showed effective knockdown of PKM2 protein expression in shRNA-mediated genetic silencing in different human HNSC cells. The representative ratios of PKM2 over internal control β-actin expression was quantified by Image J (v1.53t), and the cell used for further experiments was labelled in red. Cell growth was assayed in PKM2-silencing HNSC cells using (**C**) trypan exclusion and (**D**) MTT assays. (**E**) In vivo HNSC-bearing xenografic tumor growth analysis showed that PKM2 loss results in greater tumor mass. (**F**) PKB/Akt and (**G**) Erk proteins in PKM2-silencing HNSC cells. The representative ratios of phosphorylated PKB/Akt and Erk over total PKB/Akt and Erk was quantified by Image J (v1.53t). (**H**) Trypan blue exclusion and (**I**) MTT assays for PKM2-silencing OECM-1 cells treated with PKB/Akt inhibitor MK2206, suggesting PKB/Akt activity contribute to PKM2-mediated cell growth upregulation. Doses (μM) of inhibitor are shown in parentheses. The shLuc vector is used as a control plasmid. Data are presented as mean ± SEM (*n* ≥ 3). *** *p* < 0.001; ** *p* < 0.01; * *p* < 0.05.

The clinical impact of PKM2 in HNSC was examined in The Cancer Genomic Atlas (TCGA) and Gene Expression Omnibus (GEO) datasets using UALCAN and Kaplan–Meier plotter (K m plotter, https://kmplot.com/analysis/, accessed on 23 February 2023) web-based analysis tools. PKM2 transcripts is significantly enriched in tumorous tissues compared with their adjacent normal counterparts in most cancers (Figure S1), including HNSC samples from TCGA, GSE29330, and GSE6791 (Figure 1A). The RNA data were further confirmed by immunohistochemistry and Western blot analysis showing that, in contrast to normal oral epithelium/Normal Human Oral Keratinocytes (NHOK), PKM2 protein expression was strongly detected in 4-nitroquinoline 1-oxide (4-NQO)-induced mouse hyperplastic tongue tissues and in human oral squamous cell carcinoma (Figure S2) as well as in human HNSC cells (Figure 2A). Interestingly, to our surprise, PKM2 mRNA level exhibited a reverse correlation with tumor stage and tumor grade in GSE6791 and TCGA-HNSC datasets, respectively (Figure 1B). Moreover, a web-based tool, K m plotter [40], for survival analysis, implied that higher PKM2 expression seems to associate with poorer Overall Survival (OS) probability but is favorable for Relapse-Free Survival (RFS) probability in HNSC population (Figure 1C), indicating that PKM2 might not always act as oncogenic promoter during HNSC malignant progression and for prognosis in clinic. Further analysis revealed that age, tumor stage, and higher PKM2 expression were preferential factors correlated to poorer OS, based on univariant and multivariant analysis. In contrast, no factors showed significant contribution to RFS, as lower PKM2 expression seems correlated poorer RFS in the HNSC population (Figure 1D).

### 2.2. PKM2 Loss Facilitated HNSC Cell Growth

While PKM2 expression is enriched in most human HNSC cells, shRNA-mediated genetic knockdown was applied to establish PKM2-silencing HNSC cells (Figure 2B). Multifaceted cellular and molecular changes were evaluated in PKM2-deficient HNSC cells to gain a systemic overview of PKM2-mediated malignant changes. As Trypan blue exclusion assay indicated that cell growth is not significantly altered in response to PKM2 loss in HNSC cells (Figure 2C), MTT assay did show that PKM2 knockdown resulted in a significant increase in cell viability in human HSC3 and SAS tongue cancer cells (Figure 2D). Further analysis implied that neither cell cycling (Figure S3A) and cellular apoptosis (Figure S3B), nor cellular biomass such as genomic DNA (gDNA) and protein content (Figure S4), showed significant changes in PKM2-silening HNSC cells compared with their control counterpart. In vivo xenografic tumor growth changes in response to PKM2 loss were also examined. It was interestingly found that PKM2 downregulation led to significantly larger HNSC-bearing tumors compared with shLuc-bearing tumors (Figure 2E). At the molecular basis, HNSC is widely accepted to be one of the cancer types showing aberrant upregulated oncogenic receptor tyrosine kinase (e.g., Epidermal Growth Factor receptor, EGFR) activity [41]. EGFR hyperactivity in HNSC commonly triggers mitogenic pathways, including the RAS-ERK MAPK and AKT-PI3K-mTOR pathways [42]. Our group previously reported that the altered expression of glycolytic enzymes Hexokinase 2 (HK2) and Lactate Dehydrogenase A (LDHA) led to malignant suppression and downregulation of proto-oncogenic Akt/PKB and ERK pathway activities [43,44]. While PKM2 seems to act opposite in regulating cell growth compared with HK2 and LDHA, it is interesting to examine whether PKM2 controls cell growth via similar molecular machinery. Strikingly, Western blot analysis showed that increased PKB/Akt (Figure 2F) and ERK1/2 (Figure 2G) phosphorylation was detected in PKM2-silencing HNSC cells, suggesting that glycolytic enzymes could differently control cancerous property through the same regulatory signaling axis. Indeed, with treatment of PKB/Akt inhibitor MK2206 in HNSC cells, PKM2-mediated cell growth enhancement was suppressed in OECM-1 cells (Figure 2H,I), suggesting that the PKB/Akt pathway, at least in part, plays an important role in regulating PKM2-induced cell growth change.

### 2.3. PKM2 Silencing Led to Decreased Cell Motility in HNSC Cells

**Figure 3:**
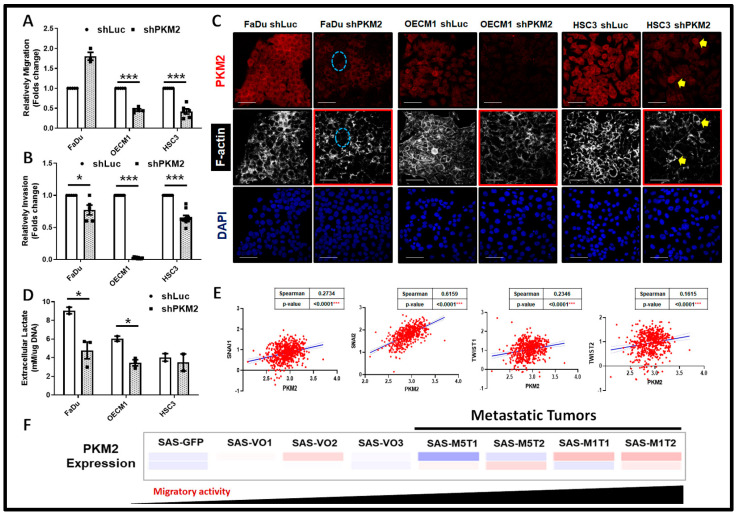
Suppression of Cell Motility in PKM2-Deficient HNSC Cells. Trans-well-based cell migration and invasion was assayed in PKM2-deficient HNSC cells. PKM2 loss led to decreased (**A**) migration and (**B**) invasion in HNSC cells. The changes in cell motility could result from (**C**) alterations of F-actin expression and (**D**) downregulation of extracellular lactate production. In (**C**), it was indicated that PKM2-silencing FaDu cells contained lower F-actin staining signals (cyan circle) as HSC3 cells with stronger PKM2 expression localized with greater F-actin staining (yellow arrows). Scale bar = 50 mm. (**E**) In the TCGA-HNSC dataset (*n* = 566), PKM2 mRNA expression is significantly positively correlated with mesenchymal markers Snail 1, Snail2/Slug, and Twist 1/2. (**F**) Microarray analysis showed a positive correlation between PKM2 mRNA expression in serial subcutaneous injected in situ (VO1/2/3) and tail vein injected metastatic (M1/M5) SAS-derived tumors (MxTx). The mRNA level is coded with different colors ranging from high (red) to low (blue). The shLuc vector is used as a control plasmid. Data are presented as mean ± SEM (*n* ≥ 3). *** *p* < 0.001; * *p* < 0.05.

The potential regulatory impact of PKM2 for HNSC cell motility was next verified using transwell-based cell migration and cell invasion assays. The results demonstrated that PKM2 deficiency resulted in significant decreased cell migration (Figure 3A) and cell invasion (Figure 3B) in HNSC cells. It was previously reported that cancer cell movement could be regulated by multiple cues, including the cytoskeletal organization [45,46], environmental lactate levels [47], and epithelial–mesenchymal transition (EMT)-associated mechanism [48] in HNSC cells. At the molecular basis, immunofluorescence staining analysis (IFA) showed that overall F-actin is less detected in PKM2-silencing HNSC cells, as PKM2 expression seems to positively correlate with F-actin abundancy (Figure 3C). Moreover, a decreased extracellular lactate production (Figure 3D) was detected predominantly in FaDu and OECM1 cells, implying that extracellular lactate level may serve as an essential factor underlying PKM2-mediated cell motility. By analyzing the TCGA-HNSC dataset, the mRNA expression of mesenchymal genes including Snail1/2 and Twist 1/2 is positively correlated with PKM2 expression (Figure 3E), revealing that EMT might be another machinery to control PKM2-mediated HNSC cell motility. The role of PKM2 in controlling in vivo tumor metastasis was further delineated in a primary and metastatic xenografic tumor model previously described [49]. Microarray analysis showed that PKM2 is enriched in highly migrating cells in this model (Figure 3F). In short, PKM2 is positively associated with cell mobility through various regulatory factors.

### 2.4. PKM2 Loss Modulated Chemosensitivity in HNSC Cells

**Figure 4:**
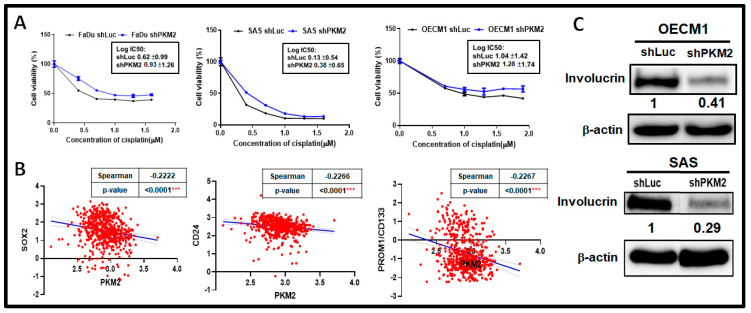
PKM2 Loss Upregulates Cisplatin Sensitivity in HNSC Cells. (**A**) Increased IC50 for clinical chemotherapeutic agent Cisplatin in PKM2-deficient HNSC cells. (**B**) In TCGA-HNSC dataset (*n* = 566), PKM2 mRNA expression is significantly reversely correlated with stemness markers Sox2, CD24, and Prom1/CD133 mRNA levels. (**C**) Suppression of cellular differentiation marker involucrin protein expression by Western blot analysis in PKM2-silencing OECM1 and SAS cells. The representative ratios of involucrin over β-actin expression were quantified by Image J (v1.53t). The shLuc vector is used as a control plasmid. Data are presented as mean ± SEM (*n* ≥ 3). *** *p* < 0.001.

The role of PKM2 in controlling chemotherapeutic efficacy was next examined. A previous study has demonstrated that PKM2 could restraint stemness related protein Oct4 thereby triggering consequent differentiation in a glioma stem cells-derived spheroid, suggesting that PKM2 could modulate cancer cell stemness/differentiation status [50]. The half maximal inhibitory concentration (IC50) of common chemotherapeutic agent Cisplatin (CDDP) in PKM2-silencing cells was measured. It was shown that a greater IC50 of CDDP was detected in PKM2-deficient HNSC cells compared with their normal counterparts (Figure 4A). As numbers of studies found that cell stemness/differentiation may contribute to chemoresistance in cancers [51,52], negative correlations between PKM2 expression and stemness markers, including Sox2, CD24, and Prom1/CD133 mRNA levels, were detected in subjects from the TCGA-HNSC dataset (Figure 4B). In contrast, the expression of the epithelial differentiation marker involucrin (IVL) was downregulated in PKM2-silencing HNSC cells compared with their counterpart (Figure 4C).

### 2.5. Metabolic Shift Was Evident in Response to PKM2 Loss

**Figure 5:**
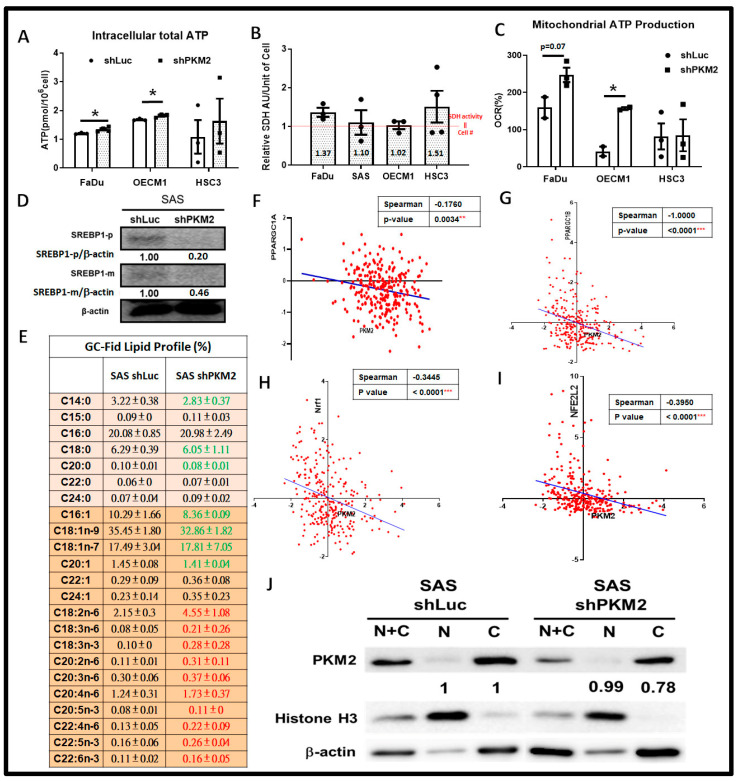
PKM2 Deficiency Elicits Metabolic Reprogramming in HNSC Cells. (**A**) Total cellular ATP level is significantly elevated in response to PKM2-silencing HNSC cells. (**B**) Mitochondrial SDH activity in single unit of PKM2-silencing HNSC cells was determined by the values of MTT levels divided by corresponding cell number. Red dotted line indicated the condition that SDH activity represents compatible cell number as in control cells. (**C**) Seahorse bioanalyzer analysis showed an upregulation of mitochondrial ATP levels in PKM2-silencing HNSC cells. (**D**,**E**) PKM2 deficiency led to decreased mature SREBP-1 level, thereby resulting in less production of de novo saturated and monounsaturated fatty acids (labelled in green) while polyunsaturated fatty acids, in contrast, were upregulated (labelled in red). By using the TCGA-HNSC dataset (*n* = 279), PKM2 mRNA expression showed a significant negative correlation with (**F**) PPARGC1A, (**G**) PPARGC1B, (**H**) Nrf1, and (**I**) NFE2L2 mRNA levels. (**J**) Westen blot analysis showed PKM2 protein expression mainly modulated in cytoplasm in shRNA-mediated PKM2-silecning SAS cells. Histon H3 was used as internal control for nuclear protein abundance. The representative ratios of PKM2 over internal control β-actin/Histon H3 expression was quantified by Image J (v1.53t). The fold changes in PKM2 expression in nuclear and cytoplasm in PKM2-silencing SAS cells was measured as PKM2 expression levels in control cells over PKM2-silencing cells. The shLuc vector is used as a control plasmid. Data are presented as mean ± SEM (*n* ≥ 3). *** *p* < 0.001; ** *p* < 0.01; * *p* < 0.05.

Based on the changes in cell malignancy in PKM2-silencing HNSC cells, it was suspected whether increased cell growth and decreased lactate secretion in response to PKM2 loss could be resulting from metabolic reprogramming. In the aspect of bioenergetics, the intracellular ATP level is upregulated in PKM2-silening HNSC cells compared to control cells (Figure 5A). This physiological change was confirmed by a decreased expression of the phosphorylated form of nutrient-sensing effectors AMP-activated protein kinase (AMPK) and The Liver Kinase B1 (LKB1) protein in PKM2-silencing OECM1 cells (Figure S5), suggesting that PKM2-silencing HNSC cells are hyperenergetic. To confirm the major ATP source in PKM2-silencing HNSC cells, numbers of metabolic assays were carried out. The results showed no evident differences for glucose uptake (Figure S6A) and for intracellular pyruvate levels (Figure S6B), implying that glycolytic activity remained unaltered. Furthermore, as we revisited the data of cell viability assays (Figure 2C,D), it was interestingly found that mitochondrial succinate dehydrogenase (SDH) activity per unit of cell, measured by MTT assay, was upregulated in PKM2-deficient cells (Figure 5B), revealing a potential metabolic shift in response to PKM2 knockdown. Seahorse metabolic assay further confirmed that PKM2 knockdown led to an increasing mitochondrial activity, including mitochondrial ATP level in FaDu and OECM1 cells (Figure 5C and Figure S7). In the aspect of cell biomass, the mRNA expression of key enzymes Transketolase (TK) and Transaldolase (TA) of the Pentose Phosphate Pathway (PPP, a metabolic pathway parallel to glycolysis that generates the nucleotide precursor ribose-5-phosphate) showed a decreasing trend in PKM2-silencing HNSC cells compared to control cells (Figure S8). For lipid anabolism, the mature form of lipogenic effector Sterol regulatory element-binding 1 (SREBP1) (Figure 5D) as well as cancer-favorable de novo biosynthesis for monounsaturated fatty acid (MUFA) were downregulated, whereas exogenous polyunsaturated fatty acid uptake was largely increased in response to PKM2 loss in SAS cells (Figure 5E). In contrast, mitochondrial β-oxidation-associated genes were mostly upregulated in PKM2-silencing HNSC cells (Figure S9). Collectively, PKM2 loss triggered a metabolic shift towards mitochondrial metabolism and reprogrammed lipid metabolism by facilitating fatty acid expenditure to support cellular malignancy. By taking advantage of the TCGA-HNSC dataset, it was also found that PKM2 mRNA expression negatively correlates with mitochondrial genes, including Peroxisome proliferator-activated receptor gamma coactivator 1 A (PPARGC1A, Figure 5F) and 1B (PPARGC1B, Figure 5G) and Nuclear factor erythroid 2-related factor 1 (Nrf1, Figure 5H) and 2 (NFE2L2, Figure 5I) in HNSC patients, further supporting the regulatory role of PKM2 expression in controlling intracellular metabolism.

In addition to pyruvate kinase activity involving the metabolic pathway, accumulating evidence also reported that PKM2 could translocate into the nucleus, serving a non-metabolic protein kinase, which phosphorylates different protein targets and contributes to multiple physiopathological processes in cancers [22]. As the above results showing PKM2 is negatively associated with HNSC malignancy seem to be contradictive to most studies, it was suspected whether shRNA-mediated silencing is effective to suppress both cytoplasmic and nuclear PKM2 expression. To address this concern, the cytoplasmic and nuclear PKM2 protein in PKM2-silencing HNSC cells were examined. The result showed that PKM2 shRNA only moderately suppressed cytoplasmic PKM2 protein expression but did not alter nuclear PKM2 expression (Figure 5J), suggesting that nuclear PKM2 may act more predominantly for tumor induction.

## 3. Discussion

**Figure 6:**
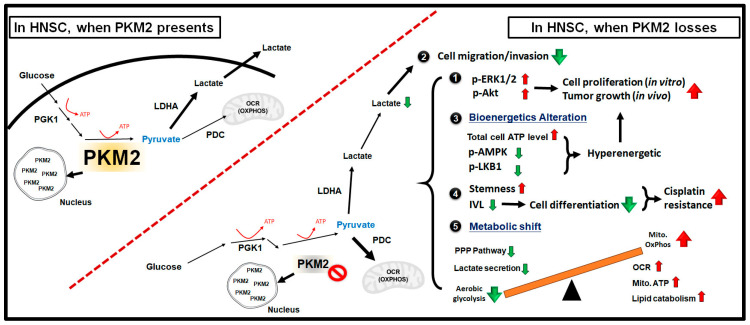
Graphic summary showing PKM2 expression modulates HNSC malignancy via metabolic reprogramming and activation of oncogenic cues. The upregulation (red arrows) and downregulation (green arrow) of physiological changes in shPKM2 treated HNSC cells compared with control cells are shown.

Due to structural and cellular location variance, it is much appreciated that PKM2 controls cancer oncogenicity in different manners. On one hand, most studies pointed out that PKM2 is enriched in cancer cells and canonically regulates glycolysis to shape the tumorous metabolic characteristics [24]; on the other hand, a number of other studies showed that PKM2 exhibited a non-canonical function as a protein kinase or a nuclear transcriptional coactivator to regulate tumor malignancy [53,54,55]. In the present study, PKM2 loss exhibited different cellular changes, including enhanced cell growth, suppression of cell motility, and elevated CDDP resistance in HNSC cells. These phenotypic alterations may result from several cellular and molecular cues. They included (i) activation of oncogenic Akt/PKB and ERK pathways, and greater intracellular ATP level resulted in increased cell growth, both in vitro and in vivo; (ii) decreasing extracellular lactate suppressed cell motility; (iii) altered cellular stemness/differentiation led to greater CDDP resistance; and (iv) induction of metabolic reprogramming away from the lactate-producing glycolysis pathway towards to mitochondrial metabolism in PKM2-silencing HNSC cells. (summarized in Figure 6).

In addition to the findings from genetic engineering animals showing PKM2 might possess tumor suppressor function [29,30], several studies also indicate that PKM2 could be dispensable for maintenance and growth in vivo [56,57]. In addition, high PKM2 expression was shown to be associated with treatment sensitivity in some types of cancer. Early studies demonstrated that PKM2 expression was downregulated in CDDP-resistant human gastric carcinoma cells compared to their parental counterparts, and suppression of PKM2 expression further increased CDDP resistance [58]. A negative correlation between oxaliplatin resistance and PKM2 mRNA levels in human colorectal cancer cells was also reported [59]. Recent findings further demonstrated that PKM2 overexpression is associated with chemosensitivity to Epirubicin and 5-Fluorouracil in breast cancers [60]. Consistent with these findings, the current results showed that PKM2 in HNSC cells may serve as a tumor-protector while PKM2-silencing HNSC cell-bearing tumors showed significantly greater tumor mass in vivo. Taking genetic variance into account, we found that no genetic mutations were detected in tested HNSC cells, whereas only a rare frequency (1.15%) of genetic mutations for the PKM2 gene were in HNSC tissues using the cBioPortal cancer genomic database (http://www.cbioportal.org, accessed on 10 Augest 2023), indicating that genetic variance is not associated with the role of PKM2 in regulating HNSC malignancy (Figure S10).

As PKM2 silencing resulted in distinct phenotypic changes in HNSC cells, the cellular bioenergetics and lipid metabolic adaptation as well as the impact of cytosol/nucleus ratio of PKM2 were noted. It is noteworthy that a gross metabolic reprogramming was found in response to PKM2 loss in HNC cells. While glycolytic activity (e.g., glucose uptake or intracellular pyruvate level) was not significantly changed, the mitochondrial-associated metabolic cues seemed predominantly upregulated, and mitochondrial ATP became a major source to fuel up PKM2-silencing HNSC cell growth. In addition, as it was widely accepted that an increased de novo fatty acid synthesis is a commonly observed feature of most cancers [61], PKM2 loss indeed suppressed cancer-favorable intracellular lipid anabolism and increased mitochondrial fatty acid catabolic gene expression in HNSC cells, suggesting that lipid utilization is crucial for PKM2-mediated tumorous properties. Another interesting finding from the current study is that shRNA-mediated gene silencing only modulated cytoplasmic PKM2 but did not inhibit nuclear PKM2 level. It was previously reported that nuclear PKM2 could phosphorylate histone H3, triggering consequent EGFR signaling for tumorigenesis and forming a positive feedback loop to enhance expression of glycolytic enzymes as well as the cancer promoting factors Oct4, HIF1α, and Signal Transducer and Activator of Transcription3 (STAT3) [62], indicating that the nucleus translocated PKM2 may act more oncogenically than its cytoplasmic counterpart. The immunohistochemical analysis in 4-NQO-induced hyperplastic tongue cancer tissues, showing that the PKM2 protein is largely detected in the nucleus (Figure S2), did support this notion. Lastly, it was also found that PKM2 loss did not significantly modulate in vitro HNSC cell growth but did significantly alter HNSC cell-bearing xenografic tumor growth. This observation suggested that PKM2 may interact with cells residing in the tumor microenvironment to regulate tumor growth in vivo. Indeed, a recent study demonstrated that ectosomal PKM2 from hepatocellular carcinoma (HCC) could remodel the tumor microenvironment and to support HCC growth [63].

Targeting PKM2 could be a promising strategy to develop anti-cancer therapeutic schemes, and several pre-clinical studies were now conducted for this purpose. Based on its multifaceted roles, PKM2 inhibitors and PKM2 activators were both developed aiming to lessen oncogenic characteristics of the PKM2 protein [22]. The strategy to inhibit PKM2 involved suppression of PKM2 expression, blocking the nuclear translocation of PKM2 and promoting the tetrameric state of PKM2, whereas PKM2 activation could often achieved by upregulating its pyruvate kinase activity [64]. To sum up, our results confirmed that PKM2 could differentially control HNSC malignancy through various regulatory machinery. In particular, it highlights the impact of metabolic plasticity in HNSC cells and the importance of systemically examining molecular/metabolic cues upon manipulation of the individual metabolic factor.

## 4. Materials and Methods

### 4.1. Chemicals, HNSC Cells, Animal and Human HNSC Tissues

Puromycin, 3-(4,5-Dimethylthiazol-2-yl)-2,5-diphenyltetrazolium bromide (MTT), and Cisplatin (CDDP) were purchased from Sigma. PKB/Akt inhibitor MK-2206 was obtained from Selleckchem. Human HNSC cell lines, including SAS and HSC3 (tongue), FaDu (hypopharyngeal), and OECM-1 (oral squamous), were obtained elsewhere [65]. The protocols of 4-NQO induction of mouse tongue cancer and the approval for accessing clinical human HNSC tissues were previously described [43].

### 4.2. HNSC Datasets

TCGA-HNSC datasets, including RNA sequencing (RNAseq) data and patient clinical information, were obtained from the UCSC Xena public data hub. The gene expression data of datasets GSE29330 [66] and GSE6791 [67] were downloaded from the Gene Expression Omnibus (GEO).

### 4.3. Establishment of PKM2-Deficient HNSC Cells

Human HNSC cells SAS, FaDu, OECM-1, and HSC3 were grown and maintained in indicated conditions [65]. The plasmids encoding small hairpin RNA (shRNA) targeting the PKM2 gene were purchased from the National RNAi Core Facility (NRCF), Academic Sinica, Taiwan (Table S1), and amplified following the standard protocol provided by NRCF. The lentiviral vectors containing shPKM2 and the control shRNA targeting Luciferase (shLuc), were generated in 293T cells. PKM2-deficient HNSC cells were cultured in medium containing 4 μg/mL puromycin for further experiments [43].

### 4.4. Cellular and Molecular Analyses

Cell viability/growth (Trypan blue exclusive and MTT assays), cell cycle, Annexin V-FITC-based cell apoptosis, transwell-based cell migration/invasion, quantitative real-time PCR (qRT-PCR), Western blot, and immunostaining analyses were performed following protocols previously described [65]. Colorimetric measurements were carried out using ELISA reader (BIO-TEK instruments, Winooski, VT, USA) and Beckman Coulter Cytomics FC500 Flow Cytometry at Instrumentation Resource Center (IRC), NYCU. The qRT-PCR analysis was processed in StepOnePlus™ Real-Time PCR System (ABI Biosystems) at IRC, NYCU. R13 (Ribosomal protein 13) was applied as an internal control, and the results are presented as Mean ± SEM. Primers for qRT-PCR analysis (Table S2) and antibodies used for Western blot and immunostaining analysis (Table S3) are listed. Image J was used to quantify protein expression. For cell fractionation experiment, the Nuclear Extraction Kit (Millipore, St. Louis, MO, USA) was used following manufacturer’s instructions.

### 4.5. Immunohistochemistry Staining Analysis

For immunohistochemistry, 4-nitroquinoline-1-oxide (4NQO, Sigma, Burlington, MA, USA) treated tongue cancer, and human HNSC tissues were stained for PKM2, as hematoxylin was used for counter staining to define cell nuclei. For immunofluorescence staining, HNSC cells were seeded on 20 × 20 mm sterile cover slides and cultured in indicated media. After treatment, cells were fixed with 4% Paraformaldehyde (PFA) for 15–20 min and stained for PKM2 and F-actin as DAPI was used for counter staining to define cell nuclei. Final images were processed using Adobe Photoshop or Olympus Fluoview Ver.3.1a Viewer.

### 4.6. XF Metabolic Assay

Measurements were made with a prototype Seahorse XF instrument. Cells were seeded in 24-well Seahorse tissue culture microplates. Approximately 45 min prior to the assay, the culture medium was exchanged with a low-buffered and non-serum RPMI medium to ensure accurate ECAR readings. For detection of acute drug responses, Oxygen Consumption Rates (OCRs) were measured followed by the sequential additions of compound solutions (Oligomycin, FCCP and Rotenone/Antimycin A).

### 4.7. Measurement of Extracellular Lactate, Intracellular Pyruvate/ATP and Glucose Uptake

Lactate colorimetric/fluorometric assay kit, ATP colorimetric/fluorometric assay kit, Pyruvate assay kit (Biovision, Waltham, MA, USA), and Glucose Uptake Cell-based Assay (Cayman Chemical, Ann Arbor, MI, USA) were carried out following manufacturer’s instructions to measure extracellular lactate, intracellular pyruvate/ATP levels, and cellular glucose uptake activity in response to PKM2 loss. Cell numbers or isolated genomic DNA contents (Bioman Scientific Co., Ltd., Taipei, Taiwan) were used to normalize lactate levels.

### 4.8. Lipidomic Analysis

For lipid profiling, cellular lipids were extracted in methanol/chloroform (*v*/*v* = 1:2) mixture and analyzed using gas chromatograph-flame ionization detector (GC-FID) at Yuanpei University of Medical Technology following the procedure previously described [68].

### 4.9. Animal Procedures

All animal studies were conducted in accordance with NYCU Institutional Animal Care and Use Committee (IACUC). Experimental procedure of 4-NQO treatment to induce mouse tongue cancer was described previously [65]. For xenografic tumor growth assay, HNSC cells with PKM2 deficiency was subcutaneously injected at the flank of nude mice from National Laboratory Animal Center, and tumor size/mass was followed for 3–4 weeks.

### 4.10. Statistical Analysis

All analyses were performed using Microsoft Excel and statistical software Prism 5 (GraphPad). All quantitative results were presented as Mean ± SEM, and significant difference was defined as the *p*-value < 0.05.

## Data Availability

Data are contained within the article and Supplementary Materials.

## Supplementary Materials

The following supporting information can be downloaded at: https://www.mdpi.com/article/10.3390/ijms242316639/s1.
