# Supplementary material for: Pyruvate Kinase Differentially Alters Metabolic Signatures during Head and Neck Carcinogenesis
Source: Int J Mol Sci. 2023 Nov 23;24(23):16639. doi: 10.3390/ijms242316639 (PMC10706023; doi:10.3390/ijms242316639)
Supplement: Supplementary file 1 [file ijms-24-16639-s001.zip › Supplemental Figures.pptx]

## Slide 1
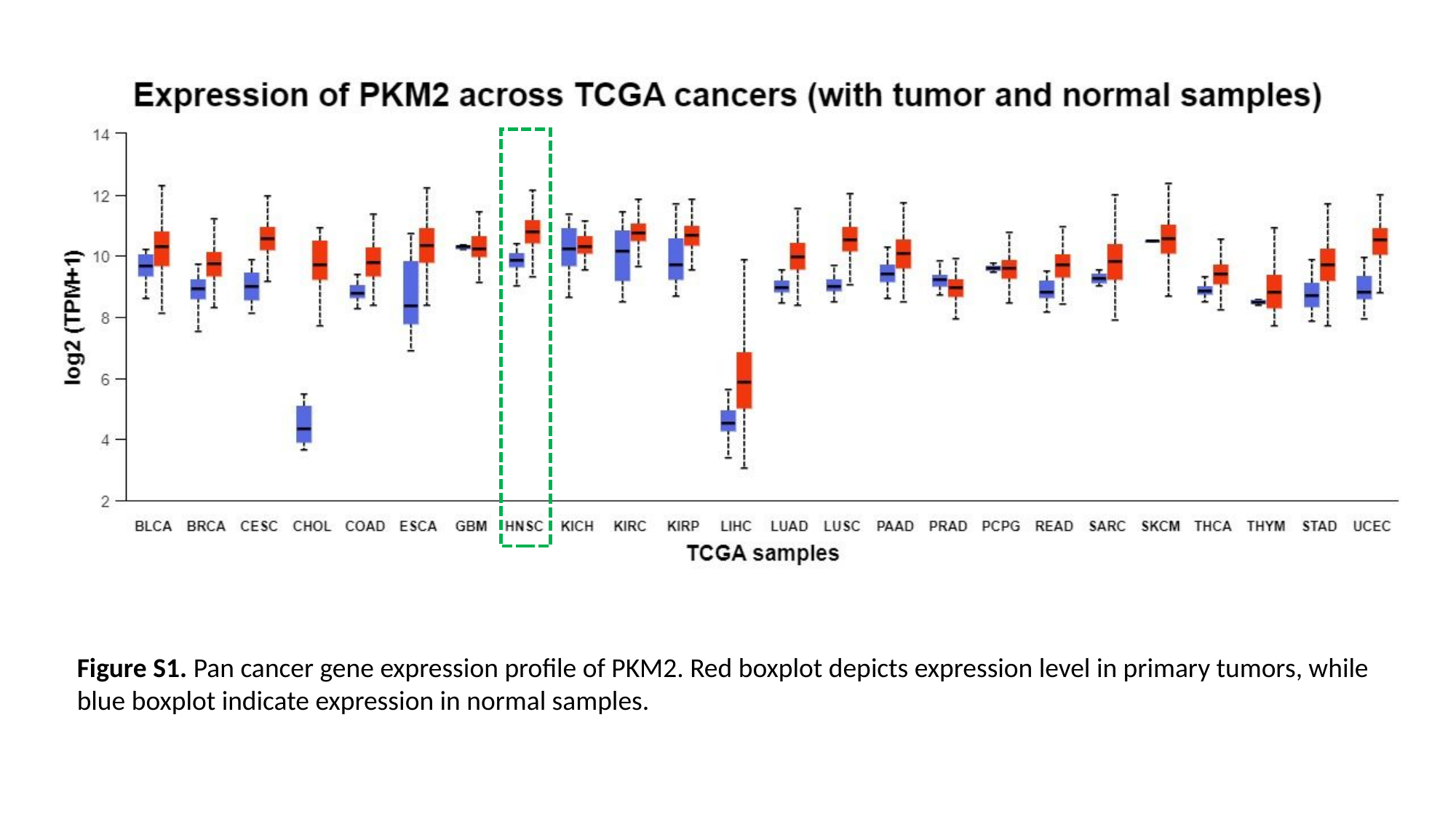

Figure S1. Pan cancer gene expression profile of PKM2. Red boxplot depicts expression level in primary tumors, while blue boxplot indicate expression in normal samples.

## Slide 2
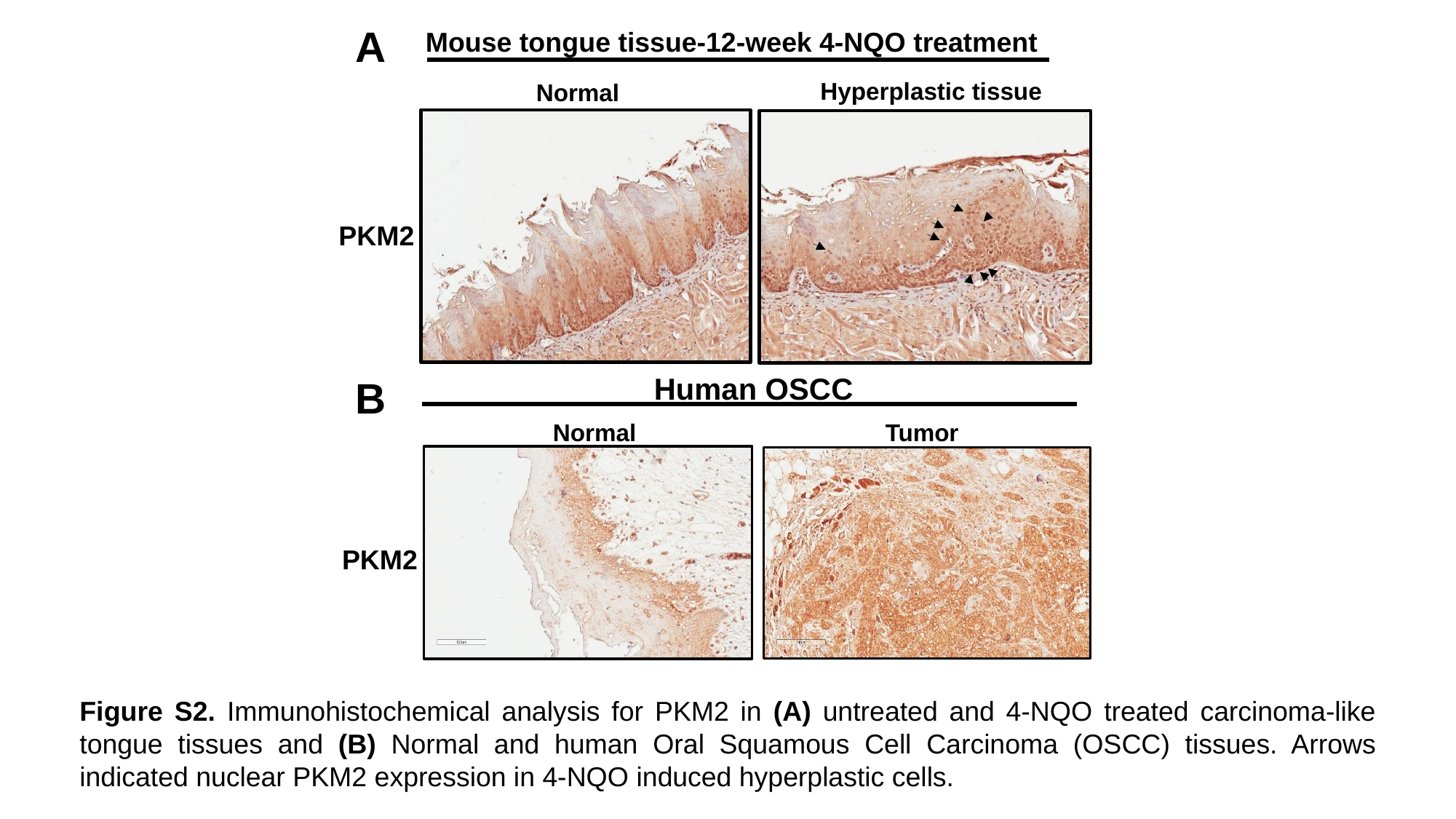

A
Mouse tongue tissue-12-week 4-NQO treatment
Hyperplastic tissue
Normal
PKM2
Human OSCC
B
Normal
Tumor
PKM2
Figure S2. Immunohistochemical analysis for PKM2 in (A) untreated and 4-NQO treated carcinoma-like tongue tissues and (B) Normal and human Oral Squamous Cell Carcinoma (OSCC) tissues. Arrows indicated nuclear PKM2 expression in 4-NQO induced hyperplastic cells.

## Slide 3
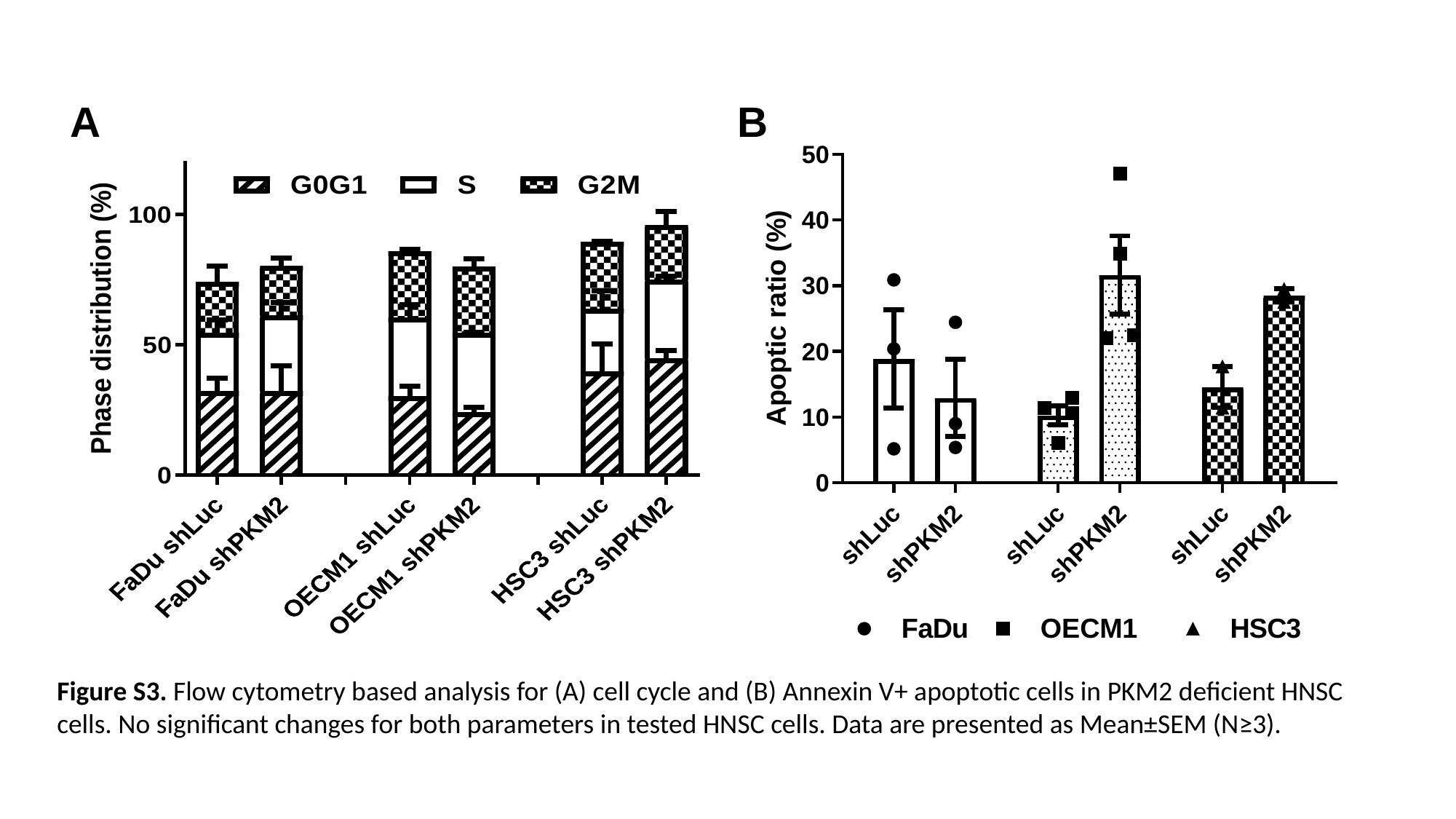

A
B
Figure S3. Flow cytometry based analysis for (A) cell cycle and (B) Annexin V+ apoptotic cells in PKM2 deficient HNSC cells. No significant changes for both parameters in tested HNSC cells. Data are presented as Mean±SEM (N≥3).

## Slide 4
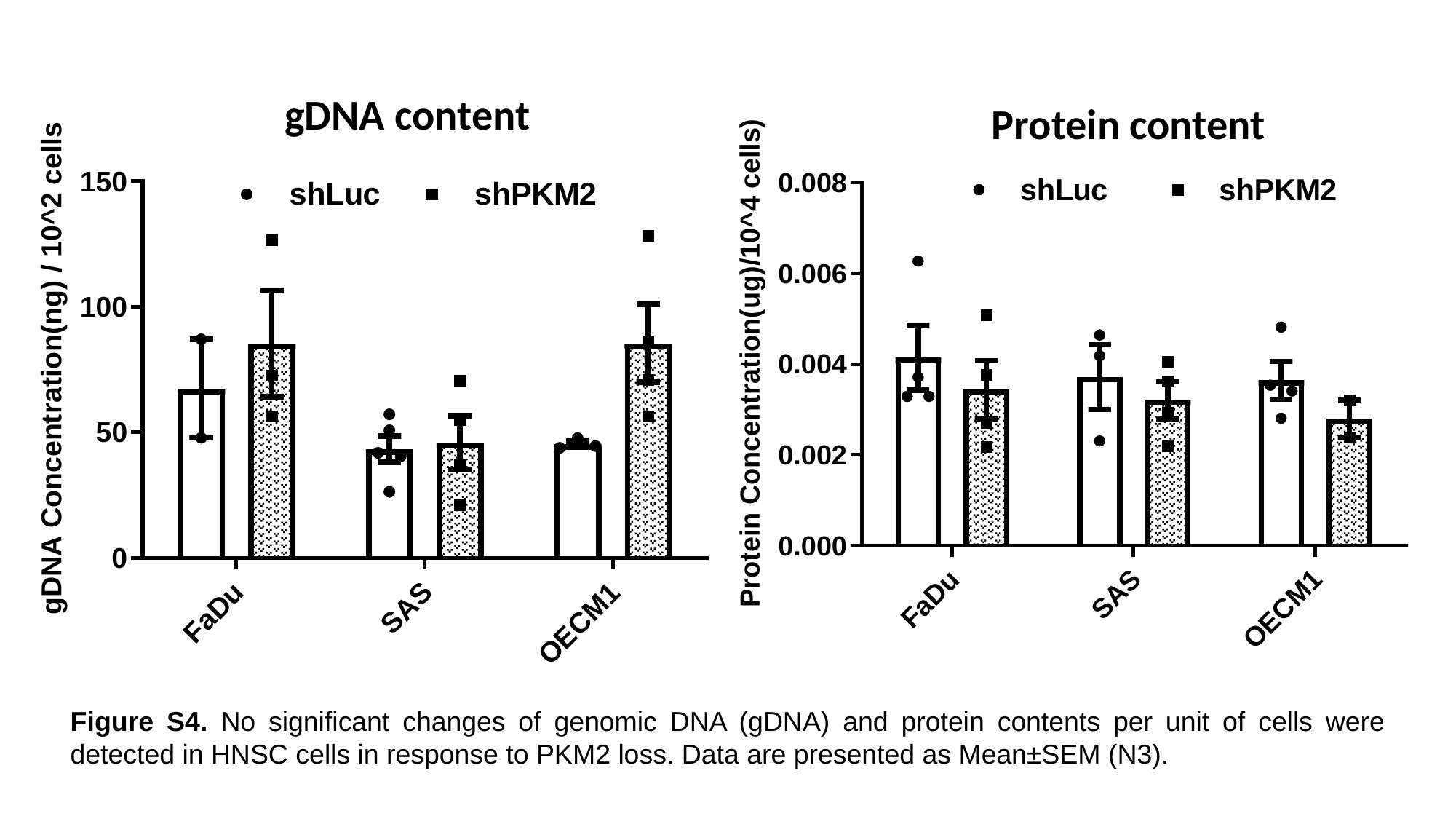

gDNA content
Protein content

## Slide 5
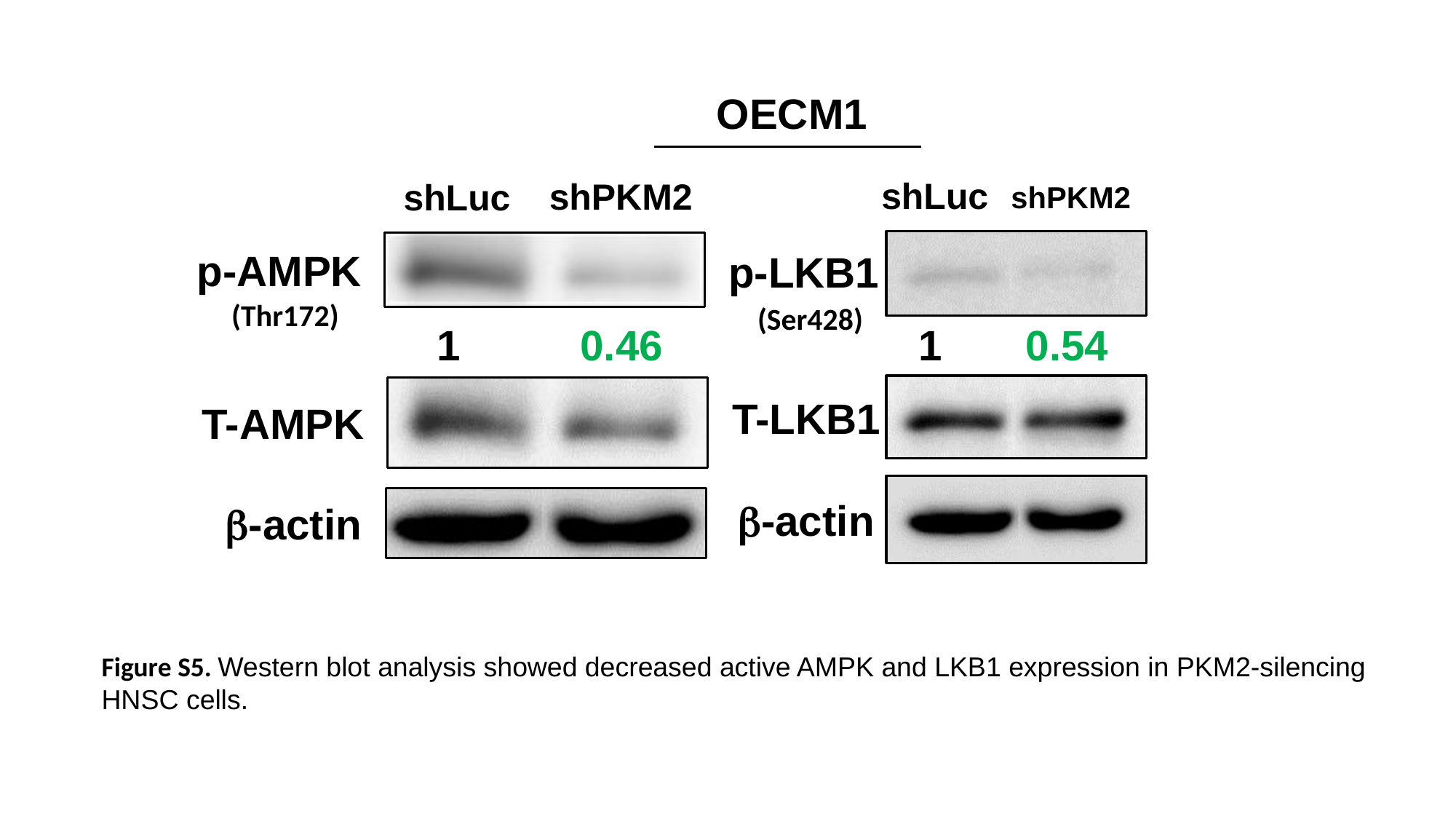

OECM1
shLuc
shPKM2
shLuc
shPKM2
p-AMPK
p-LKB1
0.46
0.54
1
1
T-LKB1
T-AMPK
b-actin
b-actin
(Thr172)
(Ser428)
Figure S5. Western blot analysis showed decreased active AMPK and LKB1 expression in PKM2-silencing HNSC cells.

## Slide 6
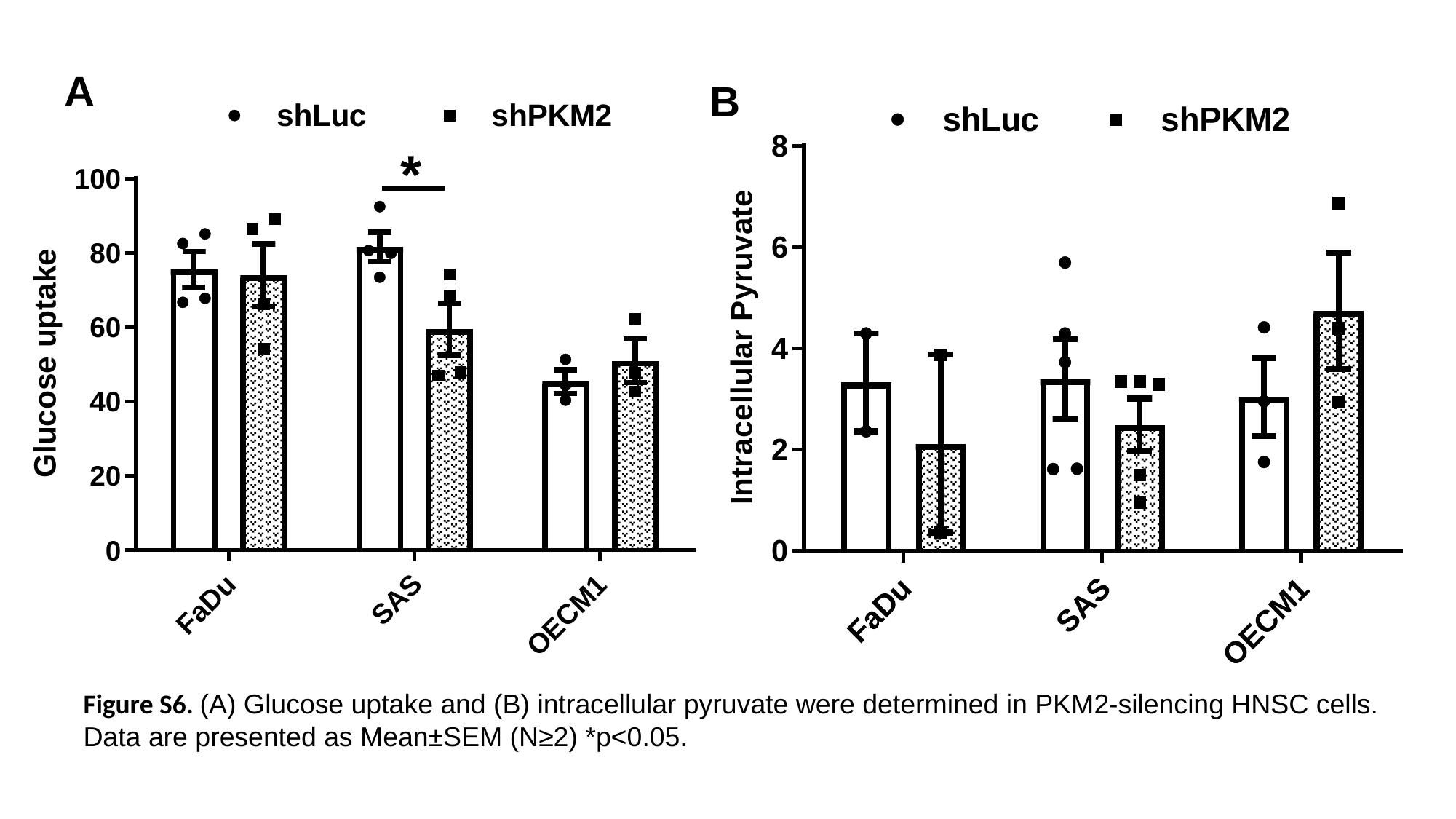

A
B
*
Figure S6. (A) Glucose uptake and (B) intracellular pyruvate were determined in PKM2-silencing HNSC cells. Data are presented as Mean±SEM (N≥2) *p<0.05.

## Slide 7
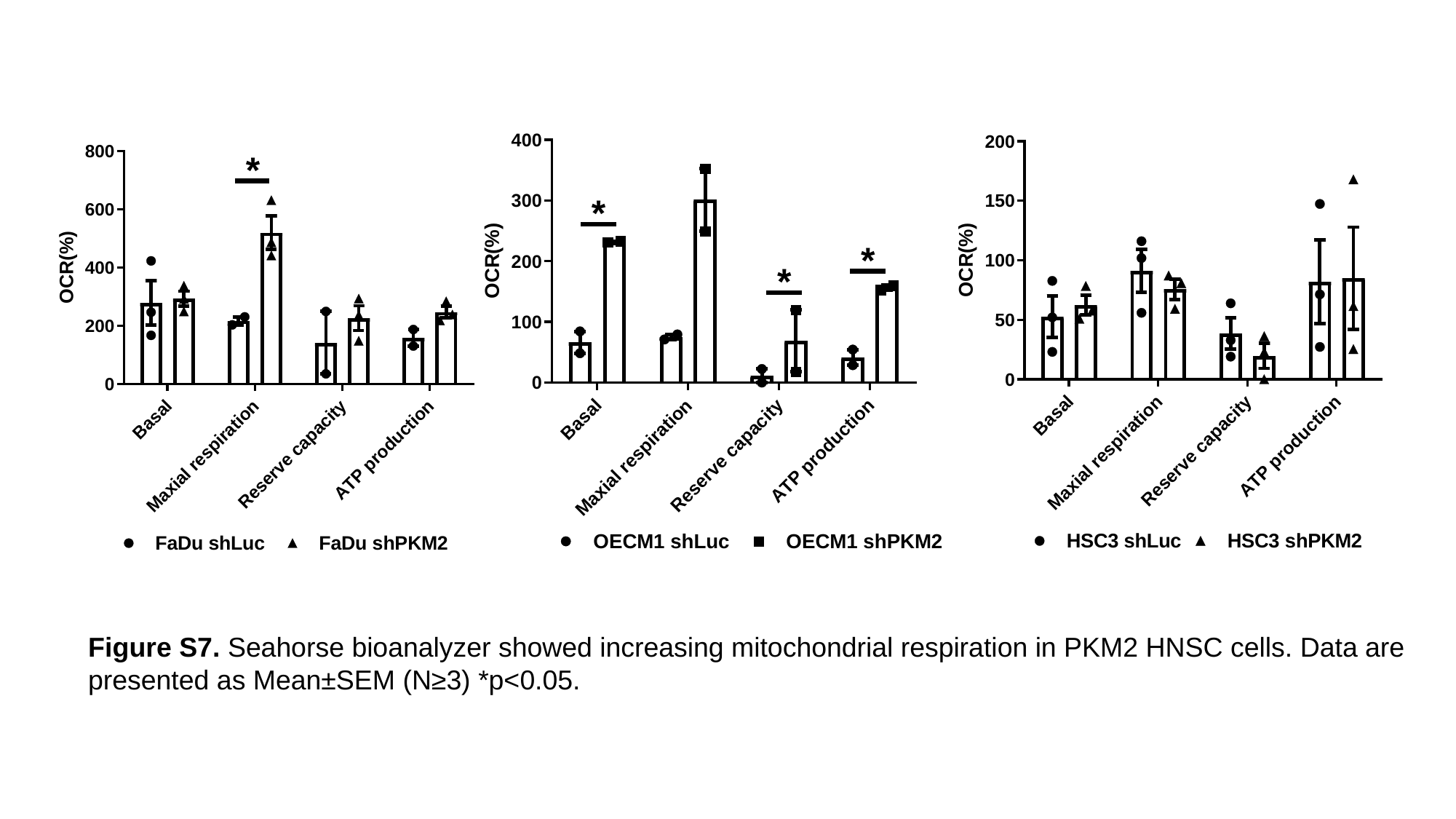

*
*
*
*
Figure S7. Seahorse bioanalyzer showed increasing mitochondrial respiration in PKM2 HNSC cells. Data are presented as Mean±SEM (N≥3) *p<0.05.

## Slide 8
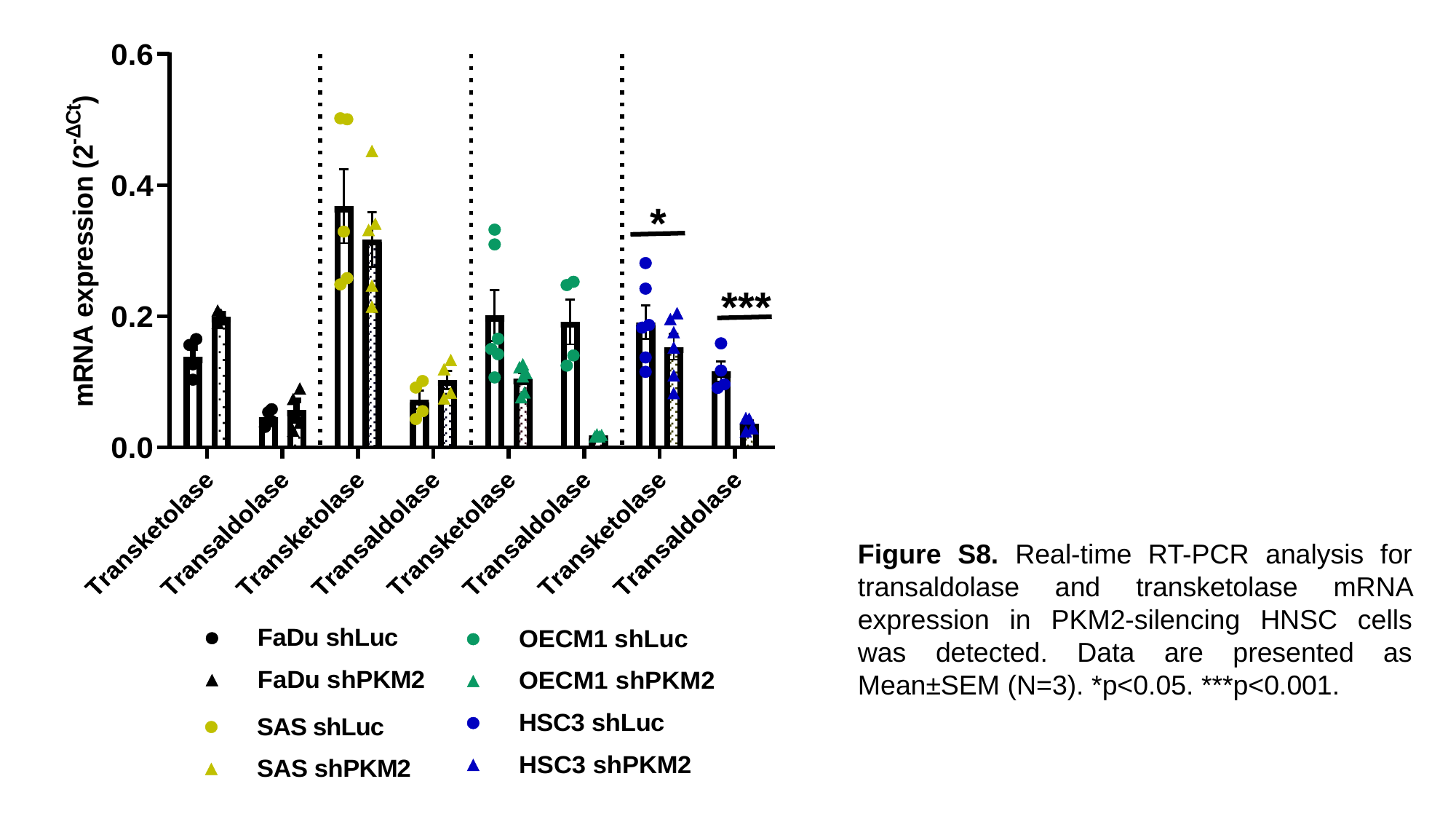

*
***
Figure S8. Real-time RT-PCR analysis for transaldolase and transketolase mRNA expression in PKM2-silencing HNSC cells was detected. Data are presented as Mean±SEM (N=3). *p<0.05. ***p<0.001.

## Slide 9
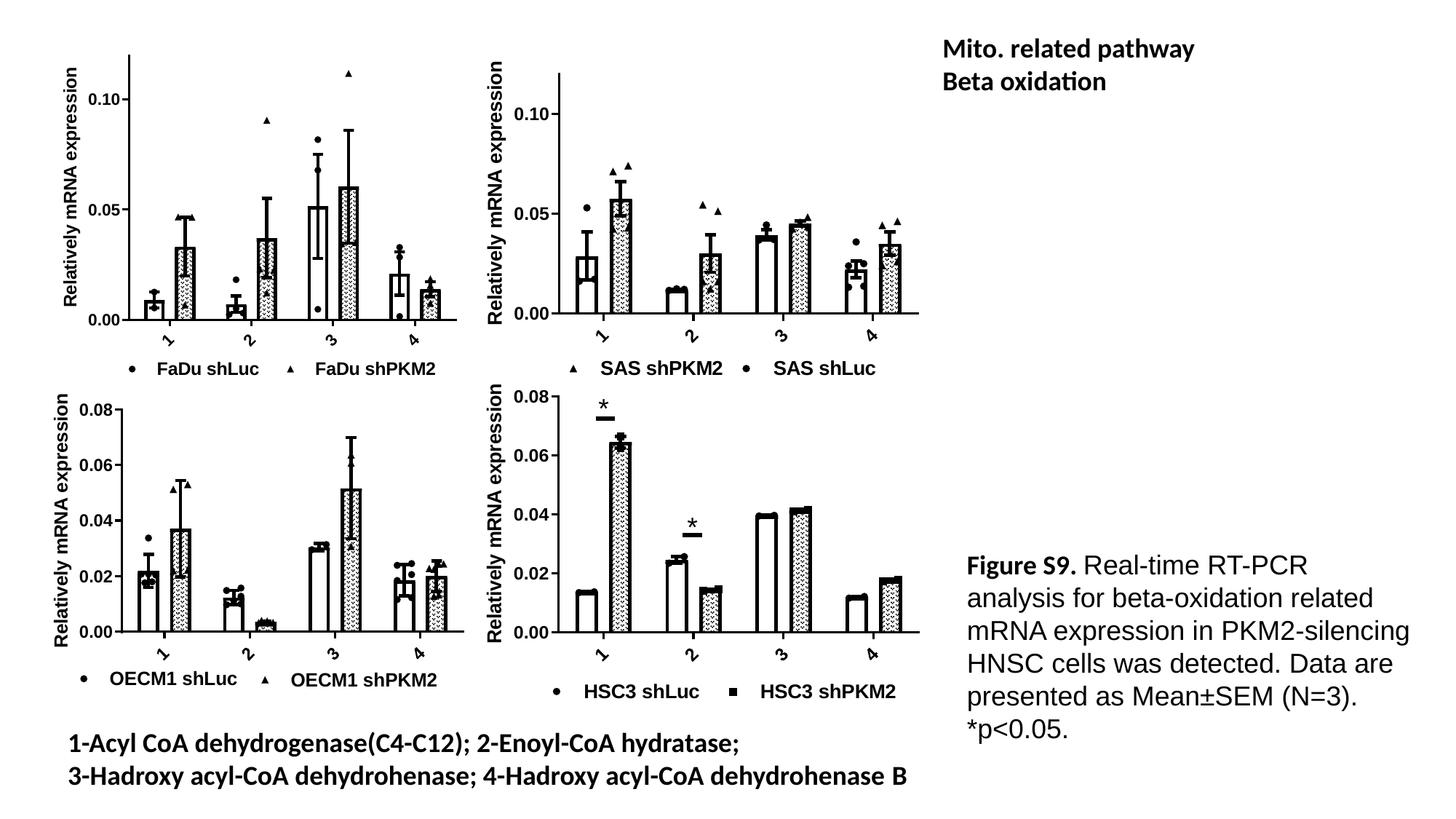

Mito. related pathway
Beta oxidation
*
*
Figure S9. Real-time RT-PCR analysis for beta-oxidation related mRNA expression in PKM2-silencing HNSC cells was detected. Data are presented as Mean±SEM (N=3). *p<0.05.
1-Acyl CoA dehydrogenase(C4-C12); 2-Enoyl-CoA hydratase;
3-Hadroxy acyl-CoA dehydrohenase; 4-Hadroxy acyl-CoA dehydrohenase B

## Slide 10
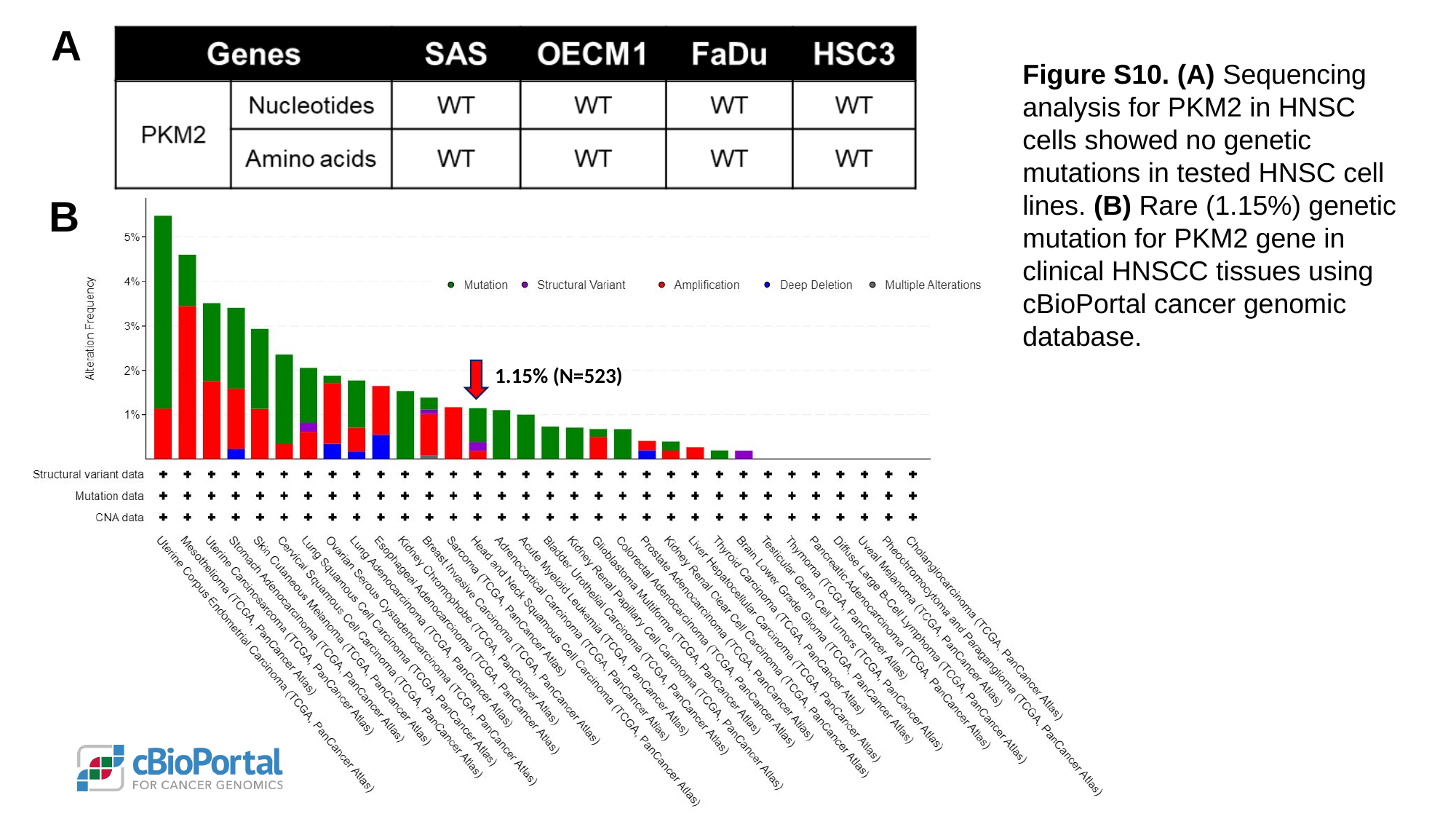

A
Figure S10. (A) Sequencing analysis for PKM2 in HNSC cells showed no genetic mutations in tested HNSC cell lines. (B) Rare (1.15%) genetic mutation for PKM2 gene in clinical HNSCC tissues using cBioPortal cancer genomic database.
B
1.15% (N=523)
