# Supplementary material for: Pyruvate Kinase Differentially Alters Metabolic Signatures during Head and Neck Carcinogenesis
Source: Int J Mol Sci. 2023 Nov 23;24(23):16639. doi: 10.3390/ijms242316639 (PMC10706023; doi:10.3390/ijms242316639)
Supplement: Supplementary file 1 [file ijms-24-16639-s001.zip › Supplemental Tables.pptx]

## Slide 1
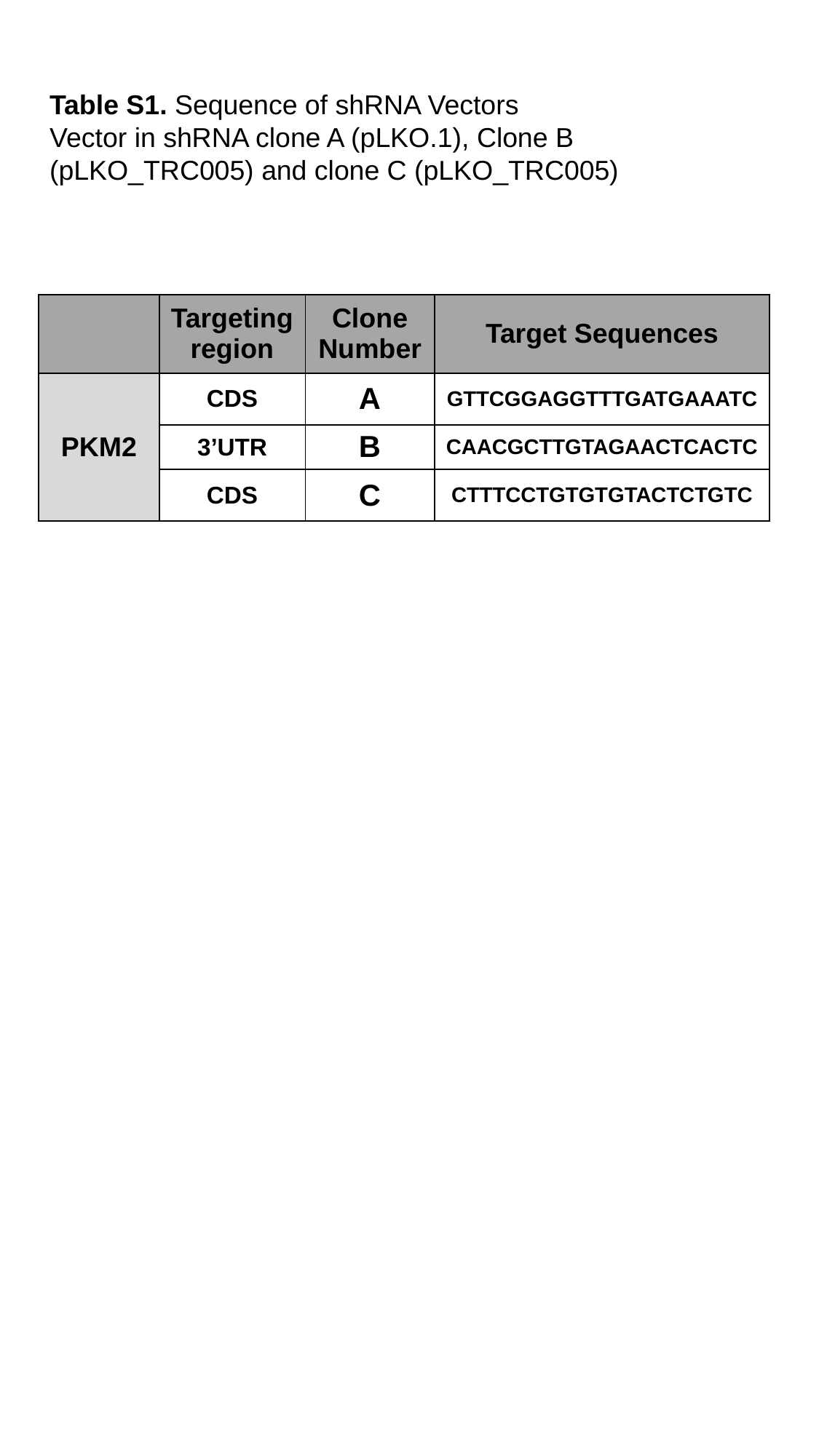

Table S1. Sequence of shRNA Vectors
Vector in shRNA clone A (pLKO.1), Clone B (pLKO_TRC005) and clone C (pLKO_TRC005)
| | Targeting region | Clone Number | Target Sequences |
| --- | --- | --- | --- |
| PKM2 | CDS | A | GTTCGGAGGTTTGATGAAATC |
| | 3’UTR | B | CAACGCTTGTAGAACTCACTC |
| | CDS | C | CTTTCCTGTGTGTACTCTGTC |

## Slide 2
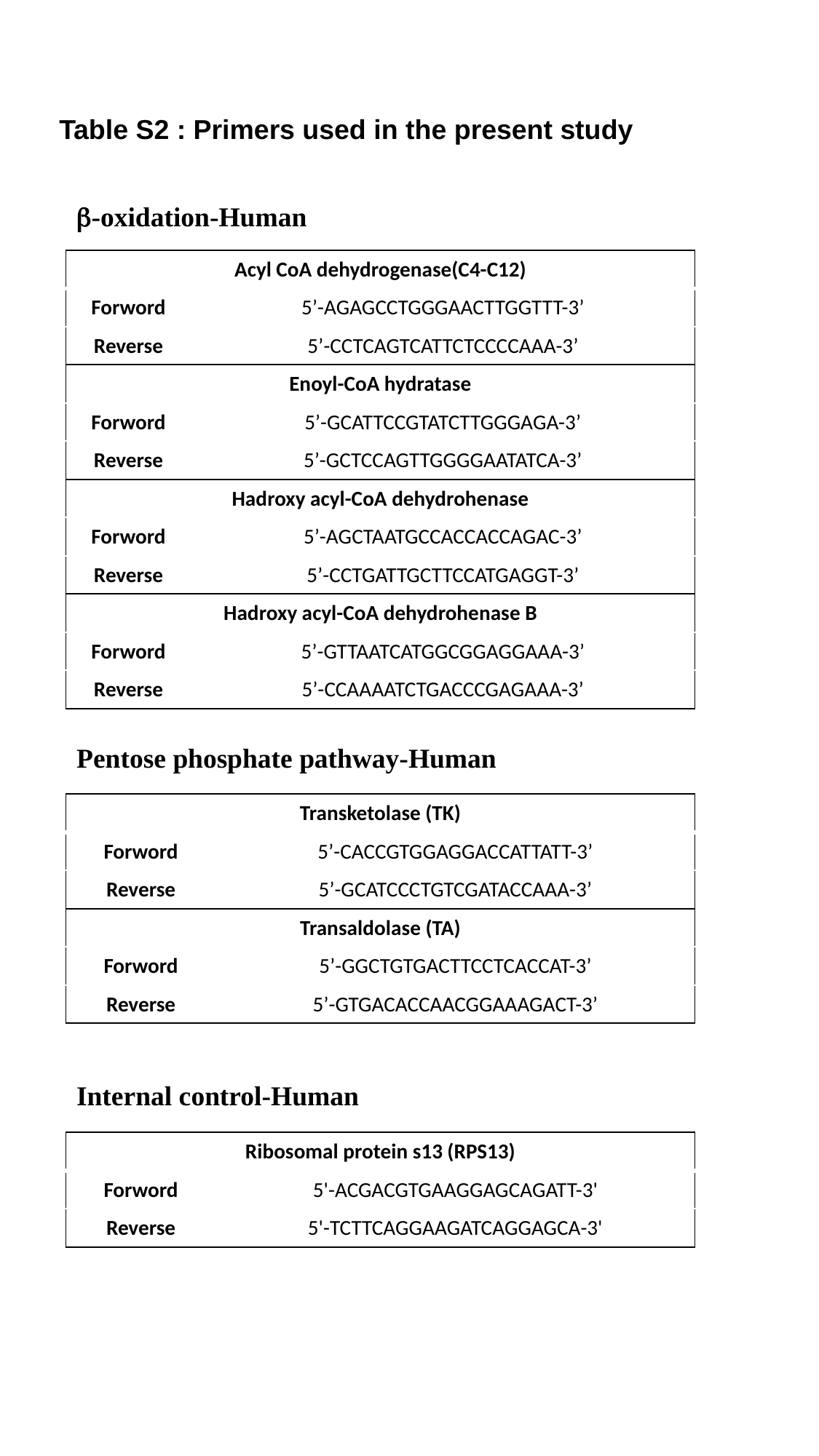

Table S2 : Primers used in the present study
b-oxidation-Human
| Acyl CoA dehydrogenase(C4-C12) | |
| --- | --- |
| Forword | 5’-AGAGCCTGGGAACTTGGTTT-3’ |
| Reverse | 5’-CCTCAGTCATTCTCCCCAAA-3’ |
| Enoyl-CoA hydratase | |
| Forword | 5’-GCATTCCGTATCTTGGGAGA-3’ |
| Reverse | 5’-GCTCCAGTTGGGGAATATCA-3’ |
| Hadroxy acyl-CoA dehydrohenase | |
| Forword | 5’-AGCTAATGCCACCACCAGAC-3’ |
| Reverse | 5’-CCTGATTGCTTCCATGAGGT-3’ |
| Hadroxy acyl-CoA dehydrohenase B | |
| Forword | 5’-GTTAATCATGGCGGAGGAAA-3’ |
| Reverse | 5’-CCAAAATCTGACCCGAGAAA-3’ |
Pentose phosphate pathway-Human
| Transketolase (TK) | |
| --- | --- |
| Forword | 5’-CACCGTGGAGGACCATTATT-3’ |
| Reverse | 5’-GCATCCCTGTCGATACCAAA-3’ |
| Transaldolase (TA) | |
| Forword | 5’-GGCTGTGACTTCCTCACCAT-3’ |
| Reverse | 5’-GTGACACCAACGGAAAGACT-3’ |
Internal control-Human
| Ribosomal protein s13 (RPS13) | |
| --- | --- |
| Forword | 5'-ACGACGTGAAGGAGCAGATT-3' |
| Reverse | 5'-TCTTCAGGAAGATCAGGAGCA-3' |

## Slide 3
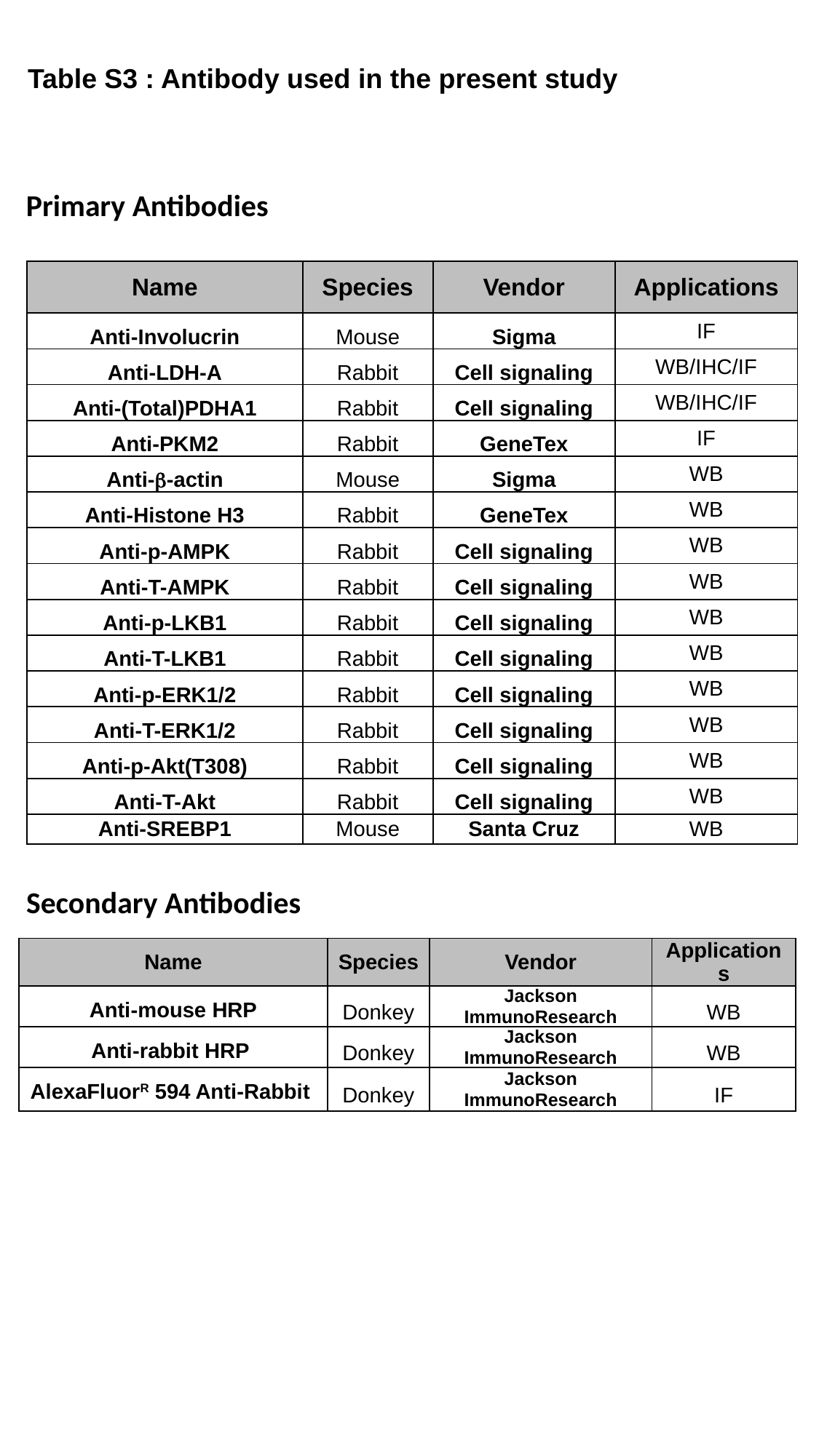

Table S3 : Antibody used in the present study
Primary Antibodies
| Name | Species | Vendor | Applications |
| --- | --- | --- | --- |
| Anti-Involucrin | Mouse | Sigma | IF |
| Anti-LDH-A | Rabbit | Cell signaling | WB/IHC/IF |
| Anti-(Total)PDHA1 | Rabbit | Cell signaling | WB/IHC/IF |
| Anti-PKM2 | Rabbit | GeneTex | IF |
| Anti-b-actin | Mouse | Sigma | WB |
| Anti-Histone H3 | Rabbit | GeneTex | WB |
| Anti-p-AMPK | Rabbit | Cell signaling | WB |
| Anti-T-AMPK | Rabbit | Cell signaling | WB |
| Anti-p-LKB1 | Rabbit | Cell signaling | WB |
| Anti-T-LKB1 | Rabbit | Cell signaling | WB |
| Anti-p-ERK1/2 | Rabbit | Cell signaling | WB |
| Anti-T-ERK1/2 | Rabbit | Cell signaling | WB |
| Anti-p-Akt(T308) | Rabbit | Cell signaling | WB |
| Anti-T-Akt | Rabbit | Cell signaling | WB |
| Anti-SREBP1 | Mouse | Santa Cruz | WB |
Secondary Antibodies
| Name | Species | Vendor | Applications |
| --- | --- | --- | --- |
| Anti-mouse HRP | Donkey | Jackson ImmunoResearch | WB |
| Anti-rabbit HRP | Donkey | Jackson ImmunoResearch | WB |
| AlexaFluorR 594 Anti-Rabbit | Donkey | Jackson ImmunoResearch | IF |
